# Novel non-carbohydrate O-GlcNAcase inhibitors with CNS drug properties as potential treatment for Alzheimer’s disease and tauopathies

**DOI:** 10.1186/1750-1326-8-S1-O17

**Published:** 2013-09-13

**Authors:** Heike Hering, Danielle Graham, Solenne Ousson, Maud Neny, Audrey Gray, Brandy Giacomozzi, John Joyce, Vikram Dutt, Michael Busch, Andrew Cameron, Leslie Liu-Bujalski, Henry Yu, Hui Tian, Mark Shearman, Anna Quattropani, Bruno Permanne, Christoph Wiessner, Dirk Beher

**Affiliations:** 1 EMD Serono Research Institute, Billerica, USA; 2 Merck KGaA, Darmstadt, Germany; 3 Asceneuron SA, Lausanne, Switzerland

## Background

Senile plaques and neurofibrillary tangles (NFTs) are characteristic hallmarks of the neuropathology of Alzheimer’s disease (AD). Numerous drug discovery efforts aimed at reducing the production or enhancing the clearance of amyloid-beta peptides, the principal component of senile plaques, have so far provided mixed results in late stage clinical development. In contrast, drug discovery programs aimed at modulating NFT pathology have been relative sparse although they present a substantial opportunity beyond AD since NFT pathology is also a major cause of tauopathies. NFTs are composed of aggregates of the microtubule associated protein, tau. Tau undergoes a variety of posttranslational modifications that can regulate its aggregation state. One such modification is the addition of O-linked N-acetylglucosamine (O-GlcNAc) moieties to serine and threonine residues by the O-linked N-acetylglucosaminyltransferase (OGT). O-GlcNAcylation of tau is a highly dynamic process driven by the addition of O-GlcNAc moieties by OGT versus the removal by O-linked-N-acetylglucosaminidase (O-GlcNAcase). Recently, it was shown that increasing O-GlcNAcylation of tau in the brain using the O-GlcNAcase inhibitor Thiamet G could decrease tau pathology in transgenic mice and stabilise against aggregation. Although Thiamet G provided the initial conceptual data in tau transgenic mice this compound is a carbohydrate substrate mimetic and as such has poor CNS drug properties. Given these limitations we initiated a drug discovery program to identify structurally distinct O-GlcNAcase inhibitor scaffolds with CNS drug properties.

## Materials and methods

To identify novel inhibitors of O-GlcNAcase we performed a high throughput screen against recombinant human enzyme. Subsequent medicinal chemistry was initiated to optimize the potency and pharmacokinetic properties of the hits. The *in vivo* pharmacodynamic response to chemically optimized inhibitors was assessed using wild-type and JNPL3 tau transgenic mice.

## Results

Novel and selective non-carbohydrate inhibitors of O-GlcNAcase were identified and optimized. Focusing on a series with a particularly good CNS profile we synthesized compound A which inhibited recombinant OGA with an IC50 of 155 nM and exhibited > 190-fold selectivity over the related enzyme, Hexosaminidase A. Single oral administration of compound A to wild-type mice resulted in a dose-dependent increase in total protein O-GlcNAcylation in the brain with a minimal effective dose between 3-10 mg/kg. Sub-chronic administration of compound A to JNPL3 tau transgenic mice resulted in an approximate 6-fold increase in the levels of O-GlcNAcylated tau in the brain as detected with our proprietary O-GlcNAc tau antibody.

## Conclusions

We have identified novel, selective and highly brain penetrant O-GlcNAcase inhibitors. These compounds have a unique non-carbohydrate backbone and show a robust pharmacodynamic response in preclinical animal models with a minimal effective dose between 3-10 mg/kg. Further chemical optimization to yield a molecule suitable for preclinical proof-of-concept in tau transgenic mice and to identify a clinical candidate for human testing is on-going.

